# Rectal diclofenac versus high-dose rectal acetaminophen in children: A randomized clinical trial

**DOI:** 10.22088/cjim.12.2.207

**Published:** 2021-03

**Authors:** Houman Hashemian, Marzie Fallah Khodadoost

**Affiliations:** 1 Pediatric Diseases Research Center , Guilan University of Medical Sciences, Rasht, Iran

**Keywords:** Acetaminophen, Diclofenac, Fever, Rectal, Suppository

## Abstract

**Background:**

Fever is the most common complaint among the children admitted to health care centers. The aim of this study was to compare the anti-pyretic effect of diclofenac and high dose acetaminophen suppository in 1 to 6 years old children.

**Methods:**

This double-blind clinical trial study was performed on 1-6-year-old children hospitalized in 17th Shahrivar Teaching Hospital, Rasht, Iran. Children were divided into two groups of 45 using a block randomization design. The first group received a high dose of acetaminophen suppository at a dose of 30 mg/kg and the second group received a diclofenac suppository at a dose of 1 mg/kg. The rectal temperature of the patients was measured using a digital thermometer at the time of drug administration, and one and three hours after that.

**Results:**

90 children were studied in two groups of 45 each. Temperature changes in the diclofenac group were significantly greater than the acetaminophen group, so from zero to 3 hours after administering diclofenac, the temperature decreased to 1.76±0.95°C. This reduction was lower in acetaminophen group (1.26±0.49°C, P=0.019).

**Conclusion:**

Both acetaminophen and diclofenac suppositories significantly reduced the rectal temperature. However, the effect of rectal diclofenac on reducing temperature is more than rectal acetaminophen.

Rectal temperature above 38 degrees Celsius is called fever. Fever is the most common complaint among the children admitted to the emergency department (1). A significant number of febrile children (about 48%) show no obvious cause despite careful evaluation (2). Infectious or non-infectious agents can release cytokines such as interleukin-1, interleukin-6, and necrotic tumor factor alpha, and interferon-gamma. These cytokines affect the body's heat regulation center in the hypothalamus, which increases body temperature (3, 4). Fever improves the immune response and slows the growth and proliferation of bacterial and viral pathogens (5). Increased patient metabolic activity, increased oxygen consumption, and increased heart and lung function are some of the effects of fever that can lead to dangerous consequences, specially in children with underlying diseases (6). There is no evidence that fever itself worsens the disease process or causes long-term neurological complications (7). Studies have shown that fever should be treated in certain cases. Fever may worsen the general condition of patients with chronic anemia or heart disease. It can also cause lung failure in patients with chronic pulmonary disease, and metabolic instability in diabetic patients and other endocrine system or metabolic diseases.

Another complication of fever in children is febrile convulsion (8, 9). However, in many cases, the fever is self-limiting or resolves with etiological treatment, if the child's rectal temperature is more than 39°C, or the child develops complications or restlessness, the use of antipyretic drugs is indicated (10, 11). Acetaminophen is one of the most commonly used analgesics and antipyretics worldwide. Because of its lower risk, it is widely used in children to relieve fever (12). Acetaminophen inhibits prostaglandin synthesis. The febrifuge effect of this drug is due to the inhibition of the effect of cyclooxygenase (COX) on body temperature regulation center (13). Acetaminophen does not inhibit COX in peripheral tissues and has no anti-inflammatory effect on peripheral tissues. For this reason, it is not classified as a non-steroidal anti-inflammatory drug (NSAID) (3, 14). Despite its beneficial effects, poisoning, renal failure and liver toxicity are the most important side effects of paracetamol that can occur due to overuse (15).

Diclofenac is a non-steroidal anti-inflammatory drug that has sedative and antipyretic effects. It is easily absorbed through the gastrointestinal tract and dissolves rapidly in an environment with a pH above five. Its half-life is about one to two hours. Diclofenac is one of the best NSAIDs that has minimal side effects when consumed rectally (16). Most studies have focused on the analgesic effect of diclofenac, did not pay much attention to the antipyretic effect of this drug, and compared it with acetaminophen. (17, 18). A study by Sharif et.al showed that diclofenac reduced fever more in the first hour after administration than acetaminophen in children. (19). 

Due to the fact that sometimes high doses of rectal acetaminophen do not lead to proper control of fever, many general physicians and pediatricians have to use diclofenac suppositories. Specifically, if the child does not have a good oral tolerance and cannot take ibuprofen syrup. Nevertheless, there are not enough studies comparing the efficacy of these two drugs in fever control. Therefore, in this study, we aimed to compare the therapeutic effect of these two drugs in the control of fever in children who referred to 17^th^ Shahrivar Hospital, Rasht, Iran. The results of this study may be helpful in choosing the right medication to control children's fever.

## Methods

**Study participants: **This double-blind controlled clinical trial was carried out on 90 children aged 1-6 years from September 2018 to June 2019 in 17^th^ Shahrivar Pediatric Teaching Hospital, Rasht-Iran. The 17^th^ Shahrivar Hospital has 120 active beds for emergency department, neonates, neonatal and pediatric intensive care units (PICU and NICU), neurology, immunology, endocrinology, nephrology, gastrointestinal, cardiology, rheumatology, hematology and infectious departments. 

Inclusion criteria include: age 1 to 6 years, weight 10 kg or more, rectal temperature of 39°C and more, and fever lasting less than 4 days. Available sampling was used. All patients who met the inclusion criteria and had been referred to the educational center from the beginning of the study until the completion of the sample size were selected. Children with a history of malignancy, gastrointestinal diseases, liver and kidney failure, neurological diseases, febrile convulsion, decreased consciousness, history of diarrhea over the past 24 hours, previous sensitivity to acetaminophen and diclofenac, anti-pyretic or antibiotic therapy during the last eight hours were excluded. The total number of samples included in the study was 121. 

However, due to recent antibiotic or acetaminophen use 22 cases, diarrhea 7 cases and seizures 2 cases were excluded. Thus, the final sample size was 90 subjects. After being informed of the purpose of the study, patients or legal guardians signed the informed consent form of the study. Then, a questionnaire was completed that assessed the child's age, sex, and weight. 

Patients were randomly assigned into either acetaminophen or diclofenac groups using a permuted-block randomization design. Twenty-two four-blocks and one two-block were used which were classified in random order. Two researchers participated in this study. One of them was responsible for sampling, completing the questionnaire, assigning patients to different treatment groups, and providing and administering medications. The second individual (pediatric assistant) was responsible for measuring the rectal temperature of the children under study before and after the intervention. 

**Figure 1:**
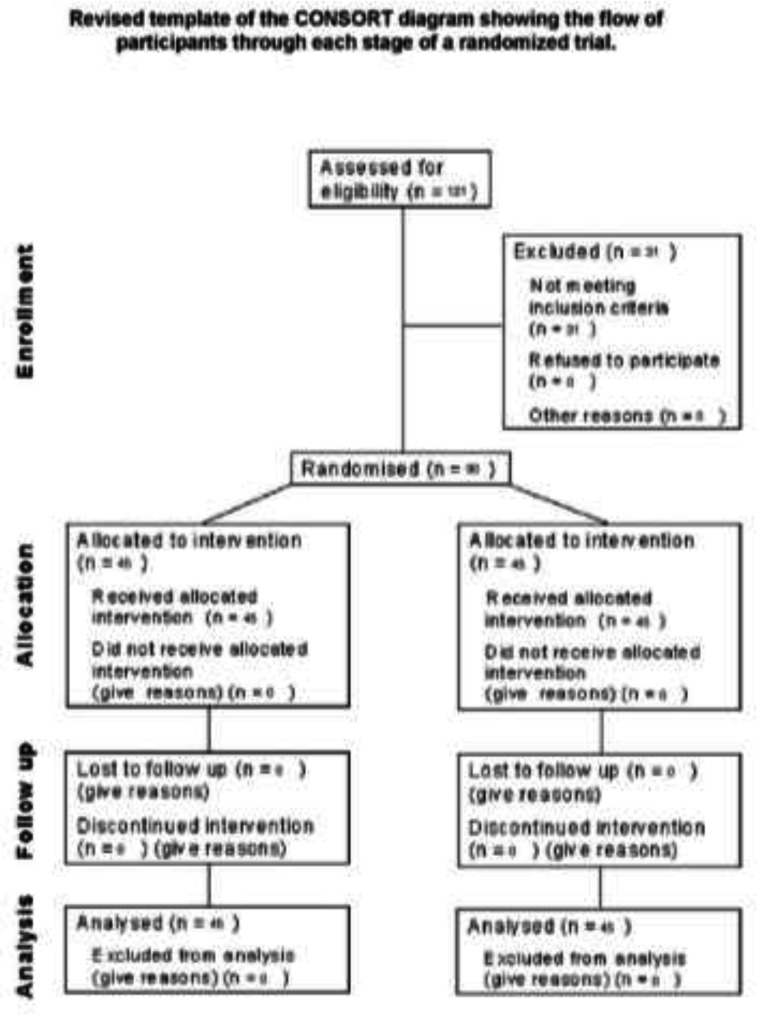
Consolidated Standards of Reporting Trials diagram depicting sample derivation

The suppositories were used for patients after exiting the envelope and preparing. Therefore, the parents and the second researcher were unaware of the type of antipyretic medicine prescribed (figure 1). The Ethics Committee of Guilan University of Medical Sciences approved this research (Approval Code: IR.GUMS.REC1397.504). This study was also registered in the Clinical Trial Registration Database in IRAN (IRCT20090909002438N3). 

According to the results of the study by Sharif et al. (19), with the test power of 95%, confidence level of 99%, 20% loss probability, and expected clinical difference of 0.5°C, the number of samples needed for each group was 45 patients treated with acetaminophen and diclofenac suppositories.

$n=\frac{{\left(z_{1-\frac{a}{2}}+z_{1}-\beta \right)}^{2}(S_{1}^{2}+S_{2}^{2})}{d^{2}}\times \frac{1}{1-f}=\frac{{\left(2.58+1.65\right)}^{2}\times (0.69^{2}+0.17^{2})}{0.5^{2}}\times \frac{1}{1-0.2}=45(\mathrm{Per Group})$

**Measuring body temperature: **The primary outcome of this study is the reduction in patient temperature after administration of antipyretic drugs. Children's body temperature was measured before drug administration and at one and three hours after that, using a digital thermometer (Express thermometer (beurer) model FT15/1 manufactured by Beurer GmbH, Germany), with a maximum error of 0.1°C. The thermometer was first disinfected with 70% ethanol, then, it was put about two cm in the child's rectum by a parent monitored by the researcher. After it was placed in the rectum for 10 seconds, the thermometer digitally displayed the child's body temperature. Although fever refers to a rectal temperature of more than 38 degrees Celsius (1), to increase the clinical significance, we considered temperature of 39°C as fever in this study.

**Treatment: **In this study, the first group received acetaminophen at a dose of 30 mg/kg body weight and the second group received diclofenac at a dose of 1 mg/kg body weight. In the present study, diclofenac 50 mg suppository manufactured by Darupakhsh-Iran Company manufacturing no. 873 and acetaminophen 125 and 325 mg suppositories manufactured by Darupakhsh-Iran Company with manufacturing nos. 304 and 622, respectively were used. The researcher cut the suppositories based on the length required and weighed with a Shenzhen Big Dipper scale (Notebook) Model 2-1108. After the parents were instructed on how to use the suppository, the drug was placed in the child's rectum by parents under the supervision of a researcher. Both groups were monitored for short-term drug side effects, such as topical effects of suppository, nausea, vomiting, itching, hives, dyspnea and hypotension and gastrointestinal symptoms (pain and bleeding) during the hospitalization period. 

**Statistical Analysis: **Data analysis was performed using IBM SPSS Statistics for Windows, Version 21.0. Armonk, NY: IBM Corp. Mean and standard deviation were calculated. The Shapiro-Wilk test was used to check the normality of the data distribution. Bonferroni independent t-test was used for group comparisons, Mann-Whitney U test for intergroup comparisons, and repeated measures ANOVA for the comparison of changes in mean scores over three time points of the study. Chi-square test was used to compare fever status in two groups in each measurement time and ANOVA was used to determine the effect of treatment methods on fever level. A p <0.05 was considered as the level of significance.

## Results

In this study, 90 children with fever were compared in two groups of acetaminophen (45 cases) and diclofenac (45 cases) in terms of temperature at three time points 0, 1 hour and 3 hours after the administration of antipyretic drug. Mean and standard deviation of age was 35.9±17.1 months. The majority of the children (64.4%) were in the age range of 3 to 6 years and only 15.6% of the children were two years or younger. The majority of the children were males (58.9%). The mean and standard deviation of the children weight was13.9±3.3 kilograms, the lowest and the highest weight were 2 and 22 kg, respectively. There was no significant difference between the two groups in terms of age, sex and weight (table 1). In the acetaminophen-treated group, body temperature decreased 1.1±0.093°C one hour thereafter, and 1.26±0.088°C three hours after administration. The difference was statistically significant. In this group, changes in temperature at one and three hours after drug administration were not statistically significant. In the group treated with diclofenac suppository, the temperature decreased 1.3±0.076 and 1.7±0.14°C, respectively, one hour and 3 hours after administration. In addition, temperature changes at one and three hours after drug administration were 0.44±0.14°C. All of these changes were statistically significant (table 2).

**Table 1:** Comparison of age, sex and weight of children studied in two treatment groups

**Patient demographic variables**			**Study group**			**P**
			**Acetaminophen suppository**	**Diclofenac suppository**	**Total**	
**Age** **(month)**	Average		36.7	35.91	35.99	0.966^*^
	Standard deviation		17.64	16.77	17.12	
	minimum		12.00	12.00	12.00	
	maximum		67.00	70.00	70.00	
**age category**	2 years and less	Number	19	13	32	0.186^**^
		Percent	42.2%	28.9%	35.6%	
	3 to 6 years	Number	26	32	58	
		Percent	57.8%	71.1%	64.4%	
	Total	Number	45	45	90	
		Percent	100.0%	100.0%	100.0%	
**sex**	Boy	Number	22	31	53	0.054^**^
		Percent	48.9%	68.9%	58.9%	
	Girl	Number	23	14	37	
		Percent	81.1%	31.1%	41.1%	
	Total	Number	45	45	90	
		Percent	100.0%	100.0%	100.0%	
**Weight (kg)**	Average		14.01	13.72	13.87	0.675^*^
	Standard deviation		3.11	3.45	3.27	
	minimum		10.0	10.0	10.0	
	maximum		21.00	22.00	22.00	

* Based on Independent T test

** Based on Chi square test

**Table 2:** Comparison of body temperature variations by study groups at study times

**Pairwise Comparisons**							
**Study group**	**Study time**	**Study time**	**Mean Difference) (I-J**	**Std. Error**	**P**_b_	**95% Confidence Interval for Difference**	
						**Lower Bound**	**Upper Bound**
Acetaminophen suppository	Start time	After 1 hour	1.113	0.093	0.000	0.882	1.345
		After 3 hours	1.260	0.088	0.000	1.042	1.478
	After 1 hour	After 3 hours	0.147	0.080	0.223	-0.053	0.346
Diclofenac suppository	Start time	After 1 hour	1.311	0.076	0.000	1.121	1.501
		After 3 hours	1.756	0.141	0.000	1.404	2.108
	After 1 hour	After 3 hours	0.444	0.140	0.008	0.096	0.793

b. Adjustment for multiple comparisons: Bonferroni

As shown in table 3, the temperature in both groups was the same at the time of drug administration and there was no statistically significant difference (P=0.744). However, this value was statistically significant between the two groups at one hour and three hours after drug administration. At both times, the mean body temperature in the acetaminophen suppository group was higher than the diclofenac.

**Figure 2:**
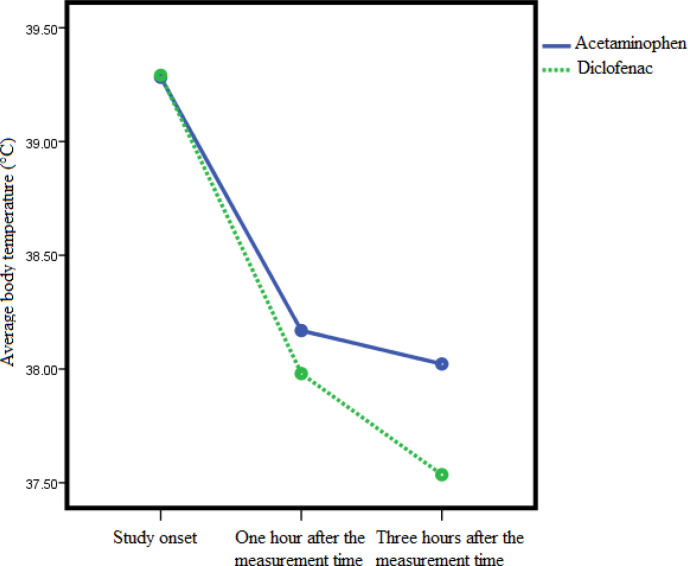
Linear graph comparing mean body temperature in the study groups by the time of study

The temperature changes had a significant downward trend (p<0.01) from the treatment onset to 1 hour, from 1 hour to 3 hours and from treatment onset to 3 hours in both acetaminophen and diclofenac suppositories. The changes in the diclofenac group were significantly greater than in the acetaminophen group, so, from zero to 3 hours after diclofenac administration, the temperature decreased (1.76±0.95°C). This decrease was lower (1.26±0.49°C) in acetaminophen group. The interaction of the effect of drug type and time based on the RM ANOVA test is plotted in figure 2.

**Table 3:** Comparison of body temperature and its variations between the studied times in the two groups

**Study criteria**		**Study group**			P*
		**Acetaminophen suppository**	**Diclofenac ** **suppository**	**Total**	
Temperature at prescription time (°C)	Average	39.28	39.29	39.29	0.744
	Standard deviation	0.34	0.38	0.36	
	minimum	39.00	39.00	39.00	
	maximum	40.50	40.50	40.50	
Temperature one hour after administration (°C)	Average	38.17	37.98	38.07	0.046
	Standard deviation	0.60	0.47	0.55	
	minimum	36.70	37.00	36.70	
	maximum	39.30	39.00	39.30	
Temperature three hours after administration (°C)	Average	38.02	37.54	37.78	0.003
	Standard deviation	0.56	0.75	0.70	
	minimum	37.00	36.30	36.30	
	maximum	39.10	70.3	39.10	
Temperature changes between the time of administration of the drug up to one hour thereafter (°C)	Average	1.11	1.31	1.21	0.033
	Standard deviation	0.62	0.54	0.58	
	minimum	0.10	0.70	0.10	
	maximum	2.70	2.70	2.70	
Temperature changes within one to three hours after drug administration (°C)	Average	0.15	0.44	0.30	0.035
	Standard deviation	0.54	0.94	0.78	
	minimum	-1.10	-1.10	-1.10	
	maximum	0.90	2.00	2.00	
Temperature changes within administration time up to three hours (°C)	Average	1.26	1.76	1.51	0.019
	Standard deviation	0.49	0.95	0.82	
	minimum	-1.10	0.40	-0.10	
	maximum	2.30	3.50	3.50	
P ** (Time effect) (In-group comparison)		0.001>	0.001>	0.001>	
P ** (drug type effect) (intergroup comparison)		0.003		Observed Power = 0.861 Eta Squared = 0.097	
P ** (effect of type and time interaction)		0.007			

*Based on Mann Whitney U test

** Based on RM ANOVA test

After controlling the temperature at baseline, temperatures were compared between the two groups one and three hours after drug administration. There was no statistically significant difference between the two groups of acetaminophen and diclofenac suppositories one hour after drug administration, but this difference was significant at three hours after administration (P=0.0019) (table 4 ). During the study period, no drug-related side effects were observed in the two groups.

**Table 4:** Comparison of body temperature between the two study groups after controlling the temperature at the start of measurement

**Temperature measurement time**	**Study group**	** Mean**	** Std. Error**	**95% Confidence Interval**		P*
				**Lower Bound**	**Upper Bound**	
Temperature one hour after administration	Acetaminophen Suppository (°C)	^a^ 38.171	0.078	38.015	38.327	0.087
	Diclofenac suppository (°C)	^a^ 37.978	0.078	37.822	38.134	
	^a^. Covariates appearing in the model are evaluated at the following values: Fever at the start of treatment (P=0.019)					
Temperature three hours after administration	Acetaminophen Suppository (°C)	38.021^a^	0.099	37.825	38.218	0.001
	Diclofenac suppository (°C)	37.536^ a^	0.099	37.340	37.733	
	^a^. Covariates appearing in the model are evaluated at the following values: Fever at the start of treatment (P=0.0316)					

^*^Based on ANOVA test

## Discussion

Fever control is essential in children with rectal temperature above 39°C as well as in high-risk children for fever, such as low age with the risk of febrile convulsion or those with underlying diseases (20). This study was designed to compare the antipyretic effect of acetaminophen and diclofenac suppositories in children. Both acetaminophen and diclofenac help lower body temperature. Acetaminophen is the first-line antipyretic drug in children, and a non-opiate analgesic (21). This drug is popular in various oral and suppository forms and is sold as an over-the-counter drug. On the other hand, diclofenac suppository is frequently prescribed by parents or physicians in cases of high and severe fever, especially in cases of oral intolerance or vomiting. However, most studies on diclofenac have focused more on the antinociceptive effect of diclofenac in adults and the ability of diclofenac as an anti-fever agent has not been widely considered (17, 18).

Our study showed that both acetaminophen and diclofenac were effective in reducing fever. Various studies have confirmed the role of acetaminophen in reducing fever and some studies have shown a better antipyretic effect for its high rectal dose (30 mg/kg body weight) (22, 23). However, this has not been confirmed in all similar studies (24). The best reported performance in the literature for this drug was a decrease of 1.07±0.16°C in the first hour after administration (25), which is close to our study finding (1.1±0.093°C). Other studies, however, have reported varying degrees of temperature reduction for acetaminophen suppositories (19, 26). These differences are difficult to justify, and many factors, such as drug dose and individual responses to medications or differences in the type of manufacturer involved can affect the performance of the drug and the results. As mentioned, this study used a high dose of rectal acetaminophen (30 mg/kg body weight). The results of our study indicated that diclofenac began its effect on lowering body temperature in children from the first hour and in average, it causes 1.7±0.14°C drop in patient temperature. Unlike acetaminophen, there have been few studies on the anti-fever effect of non-steroidal anti-inflammatory drugs, especially diclofenac, in children. The only previous study to compare the anti-fever effect of acetaminophen and diclofenac in children was in Sharif et al.’ study. They stated that diclofenac significantly reduced the body temperature of children, specially in the first hour after drug administration, compared to the usual rectal dose of acetaminophen (15 mg/kg body weight) 1.73±0.69 vs. 0.65±0.17°C respectively, p<0.001 (19). Previously, Polman et al. in a double-blind trial study of 43 children aged 2 to 10 years with a mean fever of 39.3°C showed that diclofenac significantly reduces the patient's body temperature to a normal level within 2 hours after use compared to a placebo (25).

In our study, comparing the results of acetaminophen and diclofenac suppositories in lowering body temperature shows that after taking both drugs, patients' body temperature decreases but the slope of temperature decrease at the time of study was higher in the diclofenac group than in the acetaminophen group. This means that diclofenac is more potent in lowering patients' temperature and removing fever at an equal time than acetaminophen. It is not known whether this is due to a difference in the mechanism of action of the two drugs, including the very weak effect of acetaminophen in inhibiting peripheral prostaglandin production. It should also be noted that diclofenac is rapidly absorbed from areas of the gastrointestinal tract that have a pH greater than 5 (27), while acetaminophen is better absorbed under alkaline conditions, although it is not highly dependent on the pH of the environment (28, 29). The reason for the differences in the performance of these two drugs may also be attributed to the time required to reach a maximum blood concentration. As for diclofenac, this time is approximately one hour and for acetaminophen more than two hours (30, 31). The rectal uptake of acetaminophen may also be irregular and slow due to the lipophilicity of the rectal formula or rectal pH at the time of administration (32-34). 

One of the strengths of the present study is the comparison of the therapeutic effect of acetaminophen and diclofenac suppositories, which have had few studies to date. On the other hand, the dose of the drug used for each child was calculated exactly based on his body weight. The present study also has some limitations. For example, serum levels of the drugs were not evaluated in the subjects. In other words, it was not possible to evaluate interpersonal differences in drug absorption and when the maximum serum concentration was reached. In addition, the larger sample size will definitely make the results more generalizable.

The results of this study show that both acetaminophen and diclofenac suppositories significantly lower rectal temperature. However, the effect of diclofenac suppository on reducing temperature is greater than that of acetaminophen. Therefore, rectal diclofenac can be considered in cases of high fever, especially in children at risk for fever or with oral intolerance, although further studies are needed. However, given the short half-life of diclofenac, it is very possible that the fever may return, and it is too early to recommend subsequent doses of diclofenac suppository over a fever period. Recommendations: It is recommended that similar future studies be performed on larger sample size. In addition, the possible side effects, especially if the drug is repeated, should be considered.
